# The impact of game play on dementia knowledge: A student evaluation of the Dementia Inequalities Game

**DOI:** 10.1177/14713012241306489

**Published:** 2024-12-04

**Authors:** Clarissa Giebel, Helen Marshall, Jacqui Cannon, Warren Donnellan, Heather Bullen, Elizabeth Lomas, Bridget Porritt, Anna Rees, Simon Curran, Hilary Tetlow, Mark Gabbay

**Affiliations:** Department of Primary Care & Mental Health, 4591 University of Liverpool, UK; NIHR Applied Research Collaboration North West Coast, UK; School of Allied Health and Nursing, 4591 University of Liverpool, UK; Lewy Body Society, UK; Department of Psychology, 4591 University of Liverpool, UK; School of Allied Health and Nursing, 4591 University of Liverpool, UK; SURF Liverpool, UK; Department of Primary Care & Mental Health, 4591 University of Liverpool, UK; NIHR Applied Research Collaboration North West Coast, UK

**Keywords:** dementia, education, students, game

## Abstract

**Background:** People with dementia and carers can face many barriers, or inequalities, in accessing a diagnosis or care. These barriers are unjust and can be addressed by the right interventions, to ensure that everyone receives equitable access to diagnosis and care. A lack of knowledge about dementia in the health and social care workforce is a recognised barrier. The Dementia Inequalities Game was co-produced with people with personal, professional, and voluntary sector experiences of dementia, and offers an educational tool to educate people about dementia and associated inequalities and overcome this knowledge gap in the current and future (in training) workforce. **Aims:** The aim of this study was to assess the impact of playing the co-produced Dementia Inequalities Game on knowledge about dementia and associated inequalities in health care, allied health professional, nursing and psychology students.

**Methods:** We conducted 11 game play workshops as part of regular teaching in undergraduate and postgraduate courses in psychology, nursing, occupational therapy, physiotherapy, orthoptics, and radiography at one University in the North of England. Students did not have to partake in the workshops. Participating students completed a brief before and after knowledge questionnaire about dementia and inequalities. Paired samples t-tests were used to compare ratings of knowledge of dementia and associated inequalities before and after game play.

**Findings:** Three-hundred-and-eighteen students took part in the workshops, with 312 fully completed questionnaires. The largest cohort of students (49%) were studying for a degree in nursing. Playing the game significantly increased knowledge about dementia (*p* < .001) and dementia inequalities (*p* < .001).

**Implications:** Playing the Dementia Inequalities Game is an effective tool to improve knowledge about dementia and associated inequalities in health care and psychology students. Using the game as an educational and sociable intervention in health and social care professionals is a next avenue to test.

## What does this article contribute to the wider global clinical community?

• Game play can be an effective educational tool to increase knowledge about dementia and inequalities in health, allied health, nursing and psychology students
• Playing the Dementia Inequalities Game has the potential to improve knowledge about dementia in the existing workforce

## Introduction

Dementia is a growing public health concern across the globe, with over 55 million people estimated to live with the condition (WHO, 2023). Depending on the dementia subtype, people with dementia experience different needs, which are often unmet (Miranda-Castillo et al., 2010). Their unpaid carers, including family and friends, also often experience unmet support needs, which can lead to increased levels of carer burden and poorer mental wellbeing (Mansfield et al., 2023; Sutcliffe et al., 2017). Meeting these needs is affected by a host of factors and is often determined by people with dementia’s and their carer’s background characteristics and service availability and suitability in a region (Ferguson-Coleman et al., 2020; Giebel et al., 2023, 2024a; Ketchum et al., 2022; Stephan et al., 2018). This leads to inequalities in access and use of care.

Health inequalities are differences in health outcomes based on personal, community, and infrastructure/environmental-level characteristics (Marmot et al., 2020). These inequalities based on background characteristics are unjust and can be addressed by the right set of interventions, to enable everyone to receive equal access to care and thus health and well-being. As Dahlgreen & Whitehead (2021)Dahlgren and Whitehead (2021) described, inequalities can be pictured on three different levels all affecting the health outcomes of individuals. Specifically, inequalities can occur on an individual, community and society, and general environmental, socio-economic, and cultural conditions. In the case of dementia, this rainbow model of inequalities has been modified with lived, health care, and third sector experts into the Dementia Inequalities Model (Giebel, 2024), to detail the many barriers experienced for people with dementia and their carers on an individual, community, and infrastructure level.

Lack of workforce knowledge has continually been highlighted in the literature as a key barrier to accessing suitable dementia care and receiving a correct and more timely diagnosis (McLaughlin & Laird, 2020; Wheatley et al., 2021). People below the age of 65 and those with a suspected rarer form of dementia, such as Lewy Body, Parkinson’s Disease, or fronto-temporal dementia, are repeatedly found to experience greater difficulties in receiving a dementia diagnosis (Burkinshaw et al., 2023). This is because General Practitioners and other healthcare professionals who are part of the diagnostic process can often struggle to recognise the symptoms in people below the age of 65 and those of rarer forms of dementia (Kupeli et al., 2018; O’Malley et al., 2019). Moreover, there often appears to be a lack of knowledge about dementia after the diagnosis also, and how to best support people with the condition and their carers (O’Leary et al., 2023; Weiss et al., 2020). Issues of lack of knowledge in the workforce are further amplified by a lack of integration in the UK between health and social care services, whereby once a diagnosis is made there appears to be no follow up by social care services to support the person with the condition and their family to live well supported at home. Integrated dementia care often seems to be lacking (Smith et al., 2021). All these issues create substantial barriers to people affected by dementia to access a timely and correct diagnosis and adequate care afterwards.

Educational interventions to raise knowledge about dementia vary. Time for Dementia, for example, is targeted at the future health care workforce, by ensuring regular face-to-face visits with people with dementia and their families for over 12 months (Banerjee et al., 2021; Daley et al., 2023). The programme showed positive results in improving knowledge about dementia in the students. Beyond student training programmes, educational programmes to train the existing workforce about dementia are suggested to involve active face-to-face participation, have a total duration of 8 hours, and provide guidelines to care in practice (Surr et al., 2017). These are very important parameters for providing in-depth training about dementia. However, where these programmes are missing, or even where these training programmes exist, a shorter one-off face-to-face training programme may provide fruitful to bring together the health and social care workforce in different care settings and also jointly discuss their professional caring experiences and identify any gaps in knowledge.

One way to raise awareness of dementia can be via the Dementia Inequalities Game. The board game was co-produced with 40 people living with dementia, unpaid carers, health and social care professionals, and Third Sector providers and is based on extensive research and co-production on the topic of dementia inequalities (Giebel, Hanna, et al., 2024). The roll-the-dice boardgame consists of two board halves, one focusing on pre-diagnosis and one focusing on post-diagnosis. Between two to six players can play individually or in teams and advance on the board game. Players can come across inequality and general question/activity fields. Inequality fields are linked to picking up an inequality card, which either provides a barrier and requires the player to move several steps backwards, or a facilitator, requiring the player to advance several steps. When standing on a general question/activity field, an opponent picks up a card and reads out a question about dementia, such as how many people live with the condition, what are the symptoms of Lewy Body dementia, or what are the 12 modifiable risk factors of dementia. They may also encounter an activity that is linked to memory assessments (on the pre-diagnosis board game half) or living well with dementia (on the post-diagnosis half of the board). The winner is the player or team who reaches the end first. The game was not developed for a specific population group, but for anyone interested in learning more about dementia and inequalities. Testing the game with over 50 adult members of the general public has shown significant improvements in knowledge about dementia and inequalities (Giebel, Hanna, et al., 2024). Thus, the game lends itself to being tested as an educational intervention in the current and future health and social care workforce as a next step, as it is a short education intervention which also encourages socialising when playing the boardgame. This is a notable advantage over digital or remote interventions, as the boardgame brings together different people/students/workforce to share experiences about dementia when playing.

One important aspect of the Dementia Inequalities Game is the conscious decision to create a board game, with the aim of bringing together players in person. Playing a game with others around a table facilitates sharing personal experiences and learning together, whereas digital games can be too isolating. This decision builds on the theory of social constructivism (Vygotsky, 1962), which states that learning occurs in social settings involving social interaction and interpretation.

Having successfully piloted the game in the general public previously (Giebel, Hanna, et al.., 2024), the aim of this study was to assess the impact of playing the co-produced Dementia Inequalities Game on knowledge about dementia and associated inequalities in health care, allied health professional, nursing, and psychology students. If proven effective, then the game could be recommended to be used in teaching of health, allied health, and psychology professional courses at Universities across the UK and other English-speaking countries.

## Methods

### Participants and recruitment

Undergraduate and postgraduate students in psychology, nursing, diagnostic radiography, therapeutic radiography, occupational therapy, physiotherapy, and orthoptics at the University of Liverpool, studying between December 2023 to June 2024 on one of the courses, were eligible to participate. Students were recruited within their respective courses, and could opt out of the special teaching session on the game day.

Ethical approval was obtained from the University of Liverpool ethics committee prior to study begin [ID: 12878].

### The dementia inequalities game

The game has been detailed in the Introduction and a full overview of the game is available elsewhere (Giebel, Hanna, et al.., 2024). An important focus of the game is the two separate card decks which provide the knowledge – on inequalities and on general dementia information. The inequalities card decks (one for the diagnostic journey and the first half of the board game, and one for the post-diagnostic care pathway and the second half of the board game), contain barriers or facilitators to diagnosis/ post-diagnostic care. For example, a barrier to diagnosis might focus on the fact that the GP does not recognise the player’s symptoms of dementia as they are under 65 years old, and thus the player has to move several steps backwards. This moves the player away from the finish line and indicates a barrier. In contrast, if a player picks up a facilitator card, this may detail how they have access to a link worker since their diagnosis who can help them to navigate the care system. As a result, the player can move several steps forward and be closer to the finish line.

**Figure 1.**
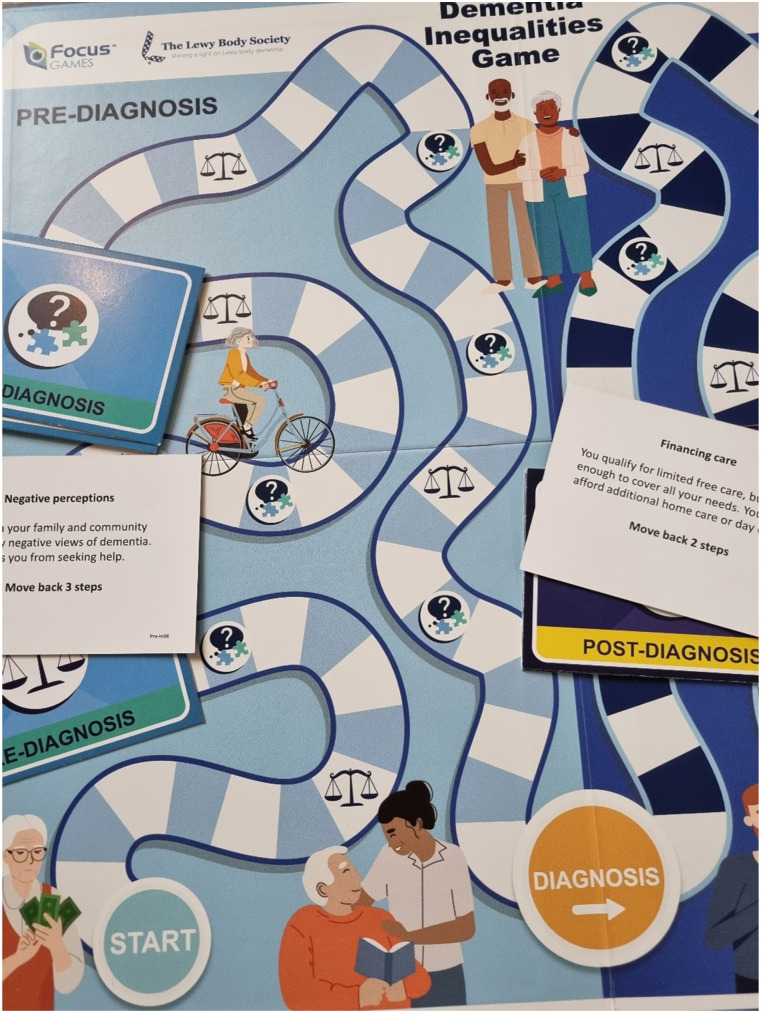
The dementia inequalities game.

In addition to inequality cards, players can also come across general activity or question fields. When landing on one of these fields, an opponent picks up a card and either asks a question (with multiple choice answers) or asks the player to engage in an activity that is linked to dementia diagnosis (i.e. memorising a series of numbers or drawing a clock, or a physical activity to stay well, such as a wall sit or singing a song). General questions about dementia, subtypes, symptomatology, and statistics have been drawn from major dementia reports, such as from Alzheimer’s Disease International (2022) and the Alzheimer’s Society (2023). Figure 1 showcases the board of the Dementia Inequalities Game.

### Data collection

Each course set up their own game play workshops between December 2023 and June 2024, and was hosted in a teaching room with group tables at the University of Liverpool. Each workshop involved students playing the game in groups of between two to six players, either as individual players or in small groups. The games were set out by the lecturers on each group table. Prior to playing the game, each student was asked to complete the written informed consent form after having had read through the study information sheet, and was asked to complete a brief pre- and post-game play questionnaire about their knowledge of dementia and associated inequalities (see Online Appendix I). The questionnaire was co-produced with two public advisors who are unpaid carers (JC, HT) and comprised of Likert scale answers for questions about participants’ knowledge about dementia and associated inequalities. Knowledge could be rated pre- and post -game play on a Likert scale ranging from 1 (‘poor’) to 5 (‘very good’). Questionnaires were anonymised and data were entered into SPSS. Workshops lasted approximately 1 hour, with the course lead offering support if required and handing all completed forms to the PI after the workshop.

There was no incentive for taking part, except for being able to play this boardgame during a teaching session.

### Data analysis

Data were analysed using paired samples t-tests, comparing pre- and post- measures on knowledge about dementia, and associated inequalities. Data were analysed using SPSS Version 29.

### Public involvement

Two public advisors were part of the study team. Both have been unpaid carers for someone with dementia previously. Both public advisors helped co-produce the questionnaire for students, and helped interpret the findings and how the game could be rolled out to students and staff across other sectors. Public advisors were reimbursed for their time according to NIHR guidance.

## Results

Between December 2023 and June 2024, 11 game play workshops were conducted with undergraduate and post-graduate students in health sciences, including nursing and psychology. A total of 318 students took part in the study, with 312 completed questionnaires. Table 1 shows the breakdown of participants per workshop and by University course. The largest cohort of students were studying for a degree in nursing (*n* = 157; 49%).

**Table 1.** Workshop participants.

#	Degree course	Number of participants
1	Radiography	14
2	Psychology	18
3	Nursing	57
4	Nursing	36
5	Occupational therapy	16
6	Physiotherapy	64
7	Therapeutic radiography	8
8	Occupational therapy	30
9	Nursing	64
10	Orthoptics	5
11	Psychology	6
Total number of participants		318

**Figure 2.**
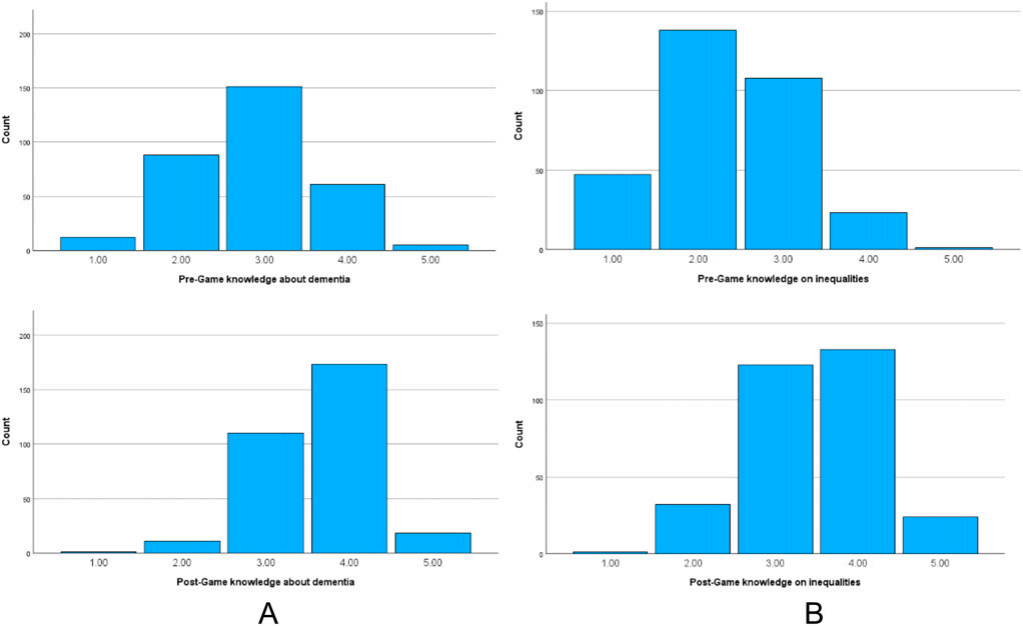
Knowledge scores before and after game play. (A) Knowledge about dementia; (B) Knowledge about dementia inequalities Scores ranged from ‘1’ (poor) to ‘5’ (very good).

Paired samples t-tests showed significant improvements in knowledge about dementia (M_pre_ = 2.9 +/−.8; M_post_ = 3.6 +/− .7; *p* < .001) and associated inequalities (M_pre_ = 2.3 +/−.8; M_post_ = 3.5 +/− .8; *p* < .001) after game play. Figure 2 shows the breakdown of score before and after game play.

## Discussion

The Dementia Inequalities Game is an effective tool to improve knowledge about dementia and inequalities and engage game players in conversation about the topic. Evaluating the impact of game play in health care and psychology students confirmed previous findings of knowledge improvements post game play in the general public (Giebel, Hanna, et al.., 2024), utilising a larger and different professional sample.

Whilst interventions to improve knowledge about dementia tend to last several hours or are spread across several weeks (i.e. Surr et al., 2017; Daley et al., 2023), engaging with the Dementia Inequalities Game is found to be an effective method of improving knowledge within a short space of time. It also allows participants to share their thoughts, knowledge, and experiences about the topic in a socially engaging way by coming together over a physical board game, as opposed to receiving training manuals or seminars. Using a game for educational purposes has been shown to be effective in other health areas such as oncology, urology, pharmaceuticals, and paediatrics (van Gaalen et al., 2021). Whilst two other one-off board games on dementia have previously been developed, these are not readily available to purchase and use in various settings as an educational tool. Niedderer et al. (2022) developed a game on mindful storytelling to improve quality of life in people with dementia, whilst Branco et al. (2017) produced one single game on activities and storytelling in two specific care facilities. Neither have been evaluated outside of their development setting and have been developed for the specific sites, without a focus on general dementia awareness raising and educating about dementia inequalities. The Dementia Inequalities Game is thus unique. Although the game is brief and cannot replace substantial training on dementia, it can be part of a multi-method approach of educating and raising awareness about dementia, which may be a more effective way of sharing knowledge and engaging the workforce, including nurses, occupational therapists, home carers, psychologists, and care home staff.

Whilst workshops involved a variety of healthcare and psychology student courses from undergraduate and postgraduate courses, students were mainly trained in health care courses, and no social care trainees were involved in the workshops. In addition, this evaluation only involved students at one University, whilst future evaluations should test the workshop method across different Universities, and different settings. However, participants are likely to have had varied experiences of dementia from personal backgrounds also, which is reflected in the pre-game knowledge scores about dementia. Future evaluations may also capture the level and type of personal experiences of dementia more clearly in a more detailed demographic background characteristics questionnaire.

This study benefits from having examined the impact of game play in more than 300 students from six degree courses, over 11 workshops, and being the only study to date to have explored the impact of playing the Dementia Inequalities Game in students and thus the future care workforce No other study had explored this to date. One possible limitation may be the short questionnaire utilised to assess dementia knowledge, albeit it had been co-produced with two unpaid carers and was purposefully developed to provide a very quick assessment of knowledge on dementia and inequalities in particular, which was not readily available before. Future work needs to explore the impact of game play in practicing health and social care professionals who are working with people with dementia and unpaid carers in different settings, including in NHS settings, care homes, day care centres, and the person with dementia’s own home, by hosting game workshops in NHS Trusts and social care organisations. Future evaluations could also include more in-depth assessments of dementia knowledge, such as via the Dementia Knowledge Assessment Scale (Annear et al., 2017). Given the effectiveness of the game on improving knowledge about dementia and inequalities, the game is being adapted into different countries and languages, including Irish, German, and Portuguese. This will further increase the reach and impact of the game on workforce and general public in different countries and cultural contexts.

### Practice implications

The Dementia Inequalities Game has significant potential to supplement teaching in psychology and allied health, but also medical degrees and social care professional qualifications. Moreover, the game can be used as an educational tool in allied health, healthcare, and social care practice. This can be achieved by hosting workshops in care homes, day care centres, or teams within NHS Trusts. Considering the short time span of game play (1 hour), the game can easily be added to ongoing professional training in the care workforces, whilst providing an engaging and social platform to share knowledge as well.

## Supplemental Material

Supplemental Material - The impact of game play on dementia knowledge: A student evaluation of the Dementia Inequalities Game Supplemental Material for The impact of game play on dementia knowledge: A student evaluation of the Dementia Inequalities Game in China by Clarissa Giebel, Helen Marshall, Jacqui Cannon, Warren Donnellan, Heather Bullen, Elizabeth Lomas, Bridget Porritt, Anna Rees, Simon Curran, Hilary Tetlow, Mark Gabbay in Dementia.
