# Supplementary material for: The impact of game play on dementia knowledge: A student evaluation of the Dementia Inequalities Game
Source: Dementia (London). 2024 Dec 4;24(8):1466–77. doi: 10.1177/14713012241306489 (PMC12508509; doi:10.1177/14713012241306489)
Supplement: Supplemental Material - The impact of game play on dementia knowledge: A student evaluation of the Dementia Inequalities Game [file sj-pdf-1-dem-10.1177_14713012241306489.pdf]

Appendix I. Pre- and post-game play questionnaire

Dementia Inequalities Game Evaluation

PRE-GAME Questionnaire

(please circle your responses and write in the provided text boxes)

1. How would you rate your knowledge about dementia? (1 – poor → 5 – very good)

   1 2 3 4 5

2. How would you rate your knowledge about inequalities in dementia?

   1 2 3 4 5

3. Do you know how many people live with dementia in the UK?

4. Do you know the most common type of dementia?

POST-GAME Questionnaire

(please circle your responses and write in the provided text boxes)

1. How would you rate your knowledge about dementia?

   1 2 3 4 5

2. How would you rate your knowledge about inequalities in dementia?

   1 2 3 4 5

3. Do you know how many people live with dementia in the UK?

4. Do you know the most common type of dementia?

5. What have you learned from playing this game?

6. Will you make any changes as a result of your learning from this game? This could be in your clinical practice or daily life.
